# Expression of miR-195 and its target gene Bcl-2 in human intervertebral disc degeneration and their effects on nucleus pulposus cell apoptosis

**DOI:** 10.1186/s13018-021-02538-8

**Published:** 2021-06-28

**Authors:** Xue-Lin Lin, Zhao-Yun Zheng, Qing-Shan Zhang, Zhen Zhang, You-Zhi An

**Affiliations:** Second Department of Spinal Surgery, The Second Hospital of Liaocheng Affiliated to Shandong First Medical University, Linqing, 252600 Shandong China

**Keywords:** miR-195, Bcl-2, Intervertebral disc degeneration, Nucleus pulposus cells, Apoptosis

## Abstract

**Objective:**

To investigate the expression of miR-195 and its target gene Bcl-2 in intervertebral disc degeneration (IVDD) and its effect on nucleus pulposus (NP) cell apoptosis.

**Methods:**

The expressions of miR-195 and Bcl-2 in NP tissues of IVDD patients were quantified by qRT-PCR and western blotting, respectively. NP cells were divided into blank group, TNF-α group, TNF-α + miR-NC group, TNF-α + siBcl-2 group, and TNF-α + miR-195 inhibitors + siBcl-2 group. Cell proliferation was detected by MTT assay, cell apoptosis evaluated by flow cytometry, and mitochondrial membrane potential (MMP) tested by JC-1 staining. Moreover, the function of miR-195 on IVDD in vivo was investigated using a puncture-induced IVDD rat model.

**Results:**

IVDD patients had significantly increased miR-195 expression and decreased Bcl-2 protein expression in NP tissues. The expression of miR-195 was negatively correlated with the expression of Bcl-2 in IVDD patients. Dual-luciferase reporter gene assay indicated that *Bcl-2* was a target gene of miR-195. In comparison with blank group, TNF-α group showed decreased cell proliferation and MMP, increased cell apoptosis, upregulated expression of miR-195, Bax, and cleaved caspase 3, and downregulated Bcl-2 protein, while these changes were attenuated by miR-195 inhibitors. Additionally, siBcl-2 can reverse the protective effect of miR-195 inhibitors on TNF-α-induced NP cells. Besides, inhibition of miR-195 alleviated IVDD degeneration and NP cell apoptosis in the rat model.

**Conclusion:**

MiR-195 was significantly upregulated in NP tissues of IVDD patients, and inhibition of miR-195 could protect human NP cells from TNF-α-induced apoptosis via upregulation of Bcl-2.

## Introduction

Intervertebral disc degeneration (IVDD) is one of the major causes of low back pain, seriously endangering the public health and bringing huge economic burdens to the whole world [1, 2]. However, the molecular mechanism of IVDD has not been clearly elucidated and multiple in vivo and in vitro factors may cause IVDD, such as genetic factors, intervertebral disc dystrophy, immunological factors, matrix metalloproteinases, inflammatory mediators, extracellular matrix (ECM) factors, apoptosis, and mechanical overload [3–5]. According to a previous study, the inner layer of intervertebral disc is nucleus pulposus (NP) ECM, which contains NP cells, proteoglycan and type II collagen [6]. IVDD occurrence has been generally considered to be associated with excessive apoptosis of NP cells [7]. Thus, inhibiting NP cell apoptosis may promote the synthesis of NP ECM to slow down the IVDD progression, which is of great significance to improve the quality of life of IVDD patients [8].

MicroRNAs (miRNA) are a class of small single-strand non-coding RNA molecules about 18–25 nt, and play critical regulatory roles in cell differentiation, proliferation and survival [9]. In recent years, miRNA is shown to involve in the development and progression of IVDD, primarily via affecting cell apoptosis, inflammatory signal response, and ECM components [10, 11]. Of note, miR-195, located at chromosome 17p13.1, belongs to the miR-15/16/195/424/497 family, which has differential roles in different diseases [12]. For example, Xiaoming Cao et al. found the increased miR-195 in osteoarthritis, which could affect the collagen synthesis in osteoarthritis progression via targeting *PTHrP* [13]. Also, miR-195 can target IKKα to enhance the proliferation and inhibit the apoptosis of human umbilical vein endothelial cells (HUVECs) treated with oxygen-glucose deprivation (OGD), as suggested by Xiao-Li Yang et al. [14]. Nevertheless, relevant studies on the role of miR-195 in IVDD are still unclear. Using the target gene prediction website, Bcl-2 was turned out to be a target gene of miR-195. Indeed, the over-expressed Bcl-2 in NP cells can significantly reduce the apoptosis of serum starvation-induced NP cells in the study of Hideki Sudo et al. [15]. Kangcheng Zhao et al. also found silencing miRNA-143 can specifically upregulate Bcl-2 expression to inhibit NP cell apoptosis [8]. Hence, we hypothesized that miR-195 may play its regulatory role in NP cell apoptosis of IVDD through the regulation of Bcl-2.

To this end, this study attempted to investigate the expression of miR-195 and its target gene *Bcl-2* in IVDD, and its possible influence in NP cell apoptosis, aiming to provide some new theoretical basis for the targeted therapy of IVDD.

## Materials and methods

### Ethics statement

The experimental protocol in this study was approved by the Ethics Committee of The Second Hospital of Liaocheng Affiliated to Shandong First Medical University. All participants and their guardians were well-informed and signed the informed consent form prior to the study. All animal studies were approved by the Institutional Animal Research Committee of The Second Hospital of Liaocheng Affiliated to Shandong First Medical University.

### Study subjects

**Fig. 1:**
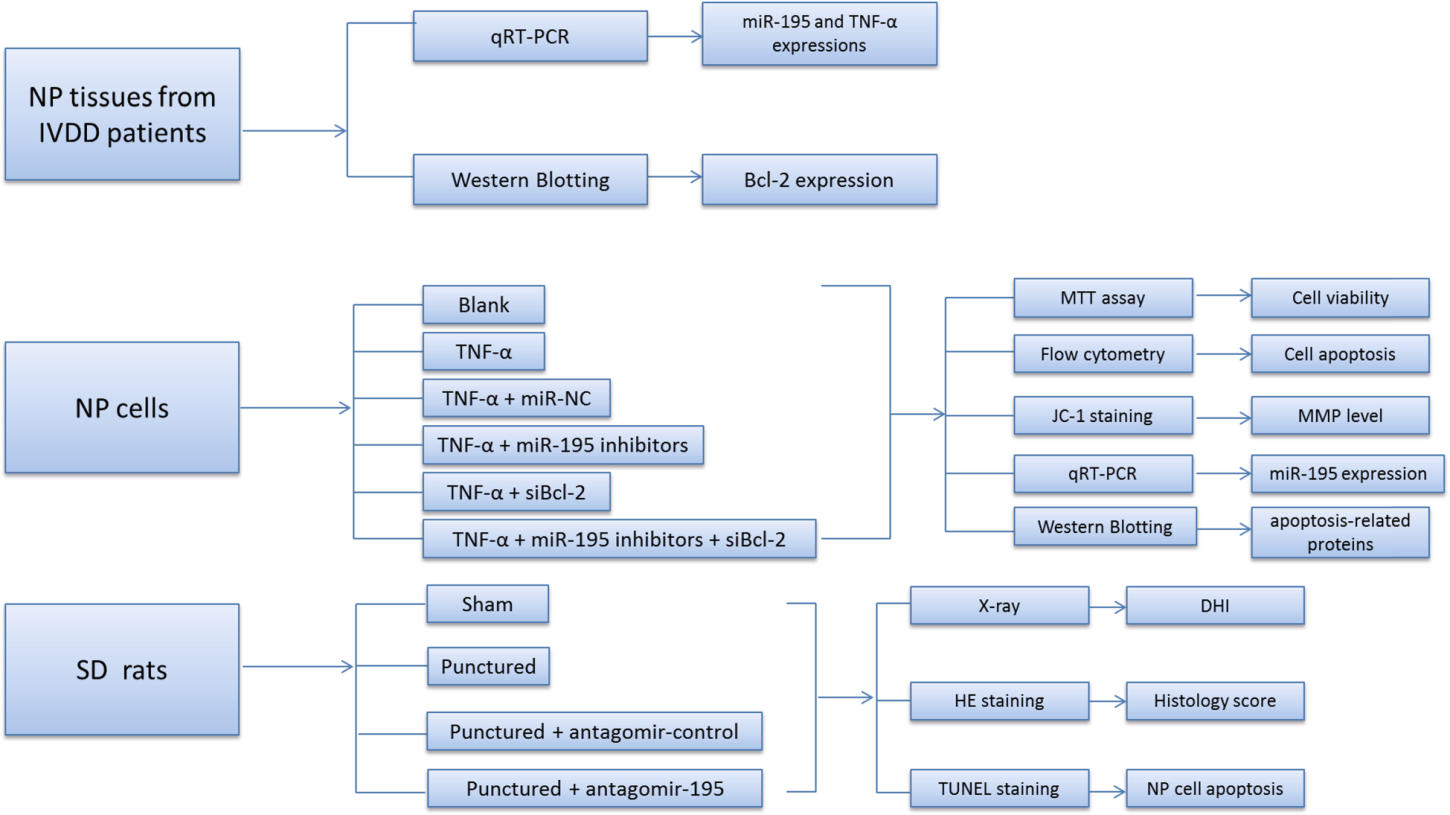
Flow chart of the experiment

From January 2018 to December 2019, 12 IVDD patients undergoing discectomy in our hospital were recruited as study subjects (Pfirrmann grade III–V, mean age 41.6 ± 8.3 years). During the same period, the normal NP tissues were obtained from 10 patients (mean age 23.4 ± 5.3 years) with idiopathic scoliosis classified as Pfirrmann grade I or II according to magnetic resonance imaging (MRI). All patients had no history of tumor, tuberculosis, diabetes, chronic infection, and autoimmune diseases. NP tissues from IVDD patients and control patients were quickly collected and stored in liquid nitrogen for later experiments, and all tissues were preserved in cell center of our hospital. Then, the expressions of miR-195 and TNF-α in NP tissues were quantified by qRT-PCR, and Bcl-2 protein expressions were detected by western blotting. Study design is summarized in Fig. 1.

### Isolation and culturing of NP cells

Human NP tissues were carefully isolated, washed three times with PBS, cut into 1 mm^3^ pieces with ophthalmic scissors, and placed in a 15-mL centrifuge tube. At the temperature of 37 °C, NP tissues were digested for 40 min in PBS solution with 0.25% trypsin (Gibco-BRL, Grand Island, NY). The digestive solution was removed, and the left tissues were washed with PBS again. Next, NP tissues were digested for 4 h in PBS solution with 0.025% type II collagen (Invitrogen, USA), followed by filtering, centrifugation, and discarding the upper supernatant. Then, the NP cells were resuspended in DMEM/F12 supplemented with 15% fetal bovine serum (FBS, Gibco-BRL), 100 μg/mL streptomycin, 100 U/mL penicillin, and 1% l-glutamine, and incubated at 37 °C in an atmosphere containing 5% CO_2_. At 80–90% confluence, cells were digested by 0.25% trypsin solution and subculture in DMEM/F12 supplemented with 15% FBS, 100 μg/mL streptomycin, 100 U/mL penicillin at 37 °C in a humidified 5% CO_2_ atmosphere. The culture medium was replaced twice every week. The second passage cells were used for subsequent experiments.

### Dual-luciferase reporter gene assay

Bioinformatics software was used to predict the target site of Bcl-2 3′UTR to bind to miR-195. Wild-type Bcl-2-Wt 3′UTR and mutant-type Bcl-2-Mut 3′UTR recombinant plasmids were constructed. One day before transfection, cells were digested by trypsin, counted (2 × 10^5^ cells/mL), and inoculated to 24-well plates. Cells were co-transfected with wild-type or mutant-type luciferase reporter gene plasmid, Renilla (pRL-TK) plasmid, and miR-195 mimic or miR-NC in accordance with the instructions of Lipofectamine 2000 kit (Invitrogen, USA). At 24 h after transaction, cells were collected to detect the luciferase activity using dual-luciferase reports gene assay kit (Promega, USA). The ratio of pGL3 firefly luciferase activity to pRL-TK Renilla luciferase activity was regarded as the relative luciferase activity. The experiment was performed three times independently to obtain the mean value.

### Grouping and transfection of NP cells

NP cells were divided into 6 groups: blank group (NP cells without any treatment), TNF-α group (NP cells treated with 20 ng/mL TNF-α for 12 h [16]), TNF-α + miR-NC group (NP cells transfected with miRNA NC prior to TNF-α treatment), TNF-α + miR-195 inhibitors group (NP cells transfected with miR-195 inhibitors prior to TNF-α treatment), TNF-α + siBcl-2 group (NP cells transfected with Bcl-2 siRNA prior to TNF-α treatment), and TNF-α + miR-195 inhibitors + siBcl-2 group (NP cells co-transfected with miR-195 inhibitors and Bcl-2 siRNA prior to TNF-α treatment). MiRNA negative control (NC), miR-195 inhibitors, and Bcl-2 siRNA were all purchased from Shanghai Genechem Co., Ltd. The miR-195 inhibitors could stably suppress the target miR-195 and are designed and optimized for miR-195 loss of function study. When reached 70–80% confluence, cells were transfected with miR-195 inhibitors/miRNA NC/Bcl-2 siRNA at a final concentration of 40 nM for transfection using Lipofectamine^TM^ 2000 (Invitrogen, USA) according to the manufacturer’s instructions.

### Quantitative reverse transcriptase polymerase chain reaction (qRT-PCR)

Total RNA in NP tissues or cells were extracted using TRIZOL agent, quantified for RNA concentration using an ultraviolet spectrophotometer, and reversely transcribed into cDNA using PrimeScript RT kit (RR014A, Takara Biomedical Technology (Beijing) Co., Ltd., China). Appropriate amount of cDNA was used as template for PCR. Primers were designed using software Primer 5.0 (Table 1) and synthesized by GenScript (Nanjing) Co., Ltd.). qRT-PCR was performed according to instructions of PCR kit (KR011A1, TIANGEN Biotech (Beijing) Co. Ltd., Beijing, China) and reaction conditions included pre-denaturation at 95 °C for 1 min and 40 cycles of denaturation at 95 °C for 10 s, annealing at 60 °C, extending for 40 s. With U6/β-actin as internal reference, 2^−△△Ct^ method was used to calculate the expression of target genes, with _△_Ct = Ct _target gene_-Ct _internal reference gene, △△_Ct=_△_Ct _experiment group_-_△_Ct _control group,_ and relative expression = 2^−△△Ct^. The experiment was performed three times independently to obtain the mean value.

**Table 1:** Primers for qRT-PCR

Gene	Primers (5′-3′)
miR-195	F: CGTAGCAGCACAGAAATATTGGC
	R: CCAGTCTCAGGGTCCGAGGTATTC
U6	F: CTCGCTTCGGCAGCACATATA
	R: ACGCTTCACGAATTTGCGTGTC
TNF-α	F: CGAGTCTGGGCAGGTCTACTTT
	R: AAGCTGTAGGCCCCAGTGAGTT
β-actin	F: GCAGAAGGAGATCACTGCCCT
	R: GCTGATCCACATCTGCTGGAA

### Western blotting

Total proteins in NP tissues or cells were extracted and quantified for protein concentration with a BCA kit. The protein samples were adjusted to the same level regarding protein content and loading volume. Polyacrylamide gel electrophoresis was performed to separate proteins, which were transferred to polyvinylidene fluoride (PVDF) membranes using semi-dry transfer system (Bio-Rad, USA). The membrane was blocked in skimmed milk at room temperature and washed with PBST buffer, before the addition of primary antibodies for 1 h reaction at room temperature, including rabbit-anti-human Bax, Bcl-2 and cleaved caspase 3, and GAPDH monoclonal antibody (all purchased from Abcam, UK). Next, the membrane was incubated for 1 h with horseradish peroxidase (HRP)-labeled goat-anti-rabbit IgG (Beijing Zhongshan Gold Bridge Biotechnology Co., Ltd.) and rinsed with PBST for 5 times × 3min. Chemiluminescence (ECL) luminescent reagent was used for the visualization of target proteins. With GAPDH protein as loading control, the software Image-pro Plus 6.0 was used to determine the relative expression of target protein. The relative expression of target protein was set as the gray value ratio of target protein to GAPDH. The experiment was performed three times independently to obtain the mean value.

### Cell viability detected by MTT assay

NP cells in each group were inoculated to 96-well plates by density of 1 × 10^4^ cells/well. When cell confluence reached 70%, 5 mg/mL MTT (3-[4,5-dimethylthiazol-2-yl]-2,5 diphenyl tetrazolium bromide) solution (ST316, Beyotime Biotechnology) was added to each well by 10 μL/well and cells were cultured at 37 °C for 4 h. After removing the upper clear supernatant, the plate was washed with PBS, followed by addition of dimethylsulfoxide (DMSO) (D5879, Sigma) by 100 μL/well and 10 min of oscillation. At 48 h after inoculation, the optical density (OD) value was determined at wavelength of 492 nm with a Microplate Reader (MK3, Thermo, Pittsburgh, PA, USA). The experiment was performed three times independently to obtain the mean value.

### Cell apoptosis detected by flow cytometry

NP cells were digested by 0.25% trypsin (without EDTA) (PYG0107, Wuhan Boster Biological Technology, Ltd., China) in a 15-mL centrifuge tube, centrifuged for 5 min at the speed of 1000 r/min, and washed three times with cold PBS. The clear supernatant was discarded. Next, 400 μL 1 × binding buffer was added to suspend cells, followed by addition of 5 μL Annexin V-FITC for 15 min staining at 4 °C in an avoidance of light, with the addition of 10 μL propidium iodide (PI) staining for 5 min at 4 °C without light. A flow cytometer was used to detect the cell apoptosis, with the excitation wavelength of 488 nm. Passband filter with the wavelength of 515 nm was used to detect FITC fluorescence, and the filter with wavelength of 560 nm was used to detect PI fluorescence. The experiment was performed three times independently to obtain the mean value.

### Detection of mitochondrial membrane potential (MMP)

In accordance with instructions of the JC-1 MMP detection kit (TIANGEN Biotech (Beijing) Co., Ltd., China), JC-1 staining solution was added to cells for 20 min of incubation at 37 °C. Next, JC-1 staining buffer was used to wash cells twice. Three hundred microliters of PBS was used to suspend cells before detection with the flow cytometer. The excitation and emission wavelength was 490 nm and 580 nm for red fluorescence, respectively, and 490 nm and 520 nm for green fluorescence, respectively. The experiment was performed three times independently to obtain the mean value.

### Establishment of rat IVDD model

Forty male Sprague-Dawley (SD) rats weighted 200–250 g were used in this study. The animals were kept at a constant room temperature of 23 ± 1 °C on a 12-h light/dark cycle and had free access to food and tap water. Rats (n = 40) were randomly divided into four groups (n = 10 per group): sham group, punctured group, punctured + antagomir-control, and punctured + antagomir-195 group. A rat model of IVDD was established using the annulus fibrosus needle puncture method [17]. In brief, the rats were anesthetized by intraperitoneal injection of 10 mg/kg xylazine and 90 mg/kg ketamine hydrochloride. Subsequently, the syringe needle was inserted into the coccygeal discs C6–C7 in a vertical direction, and then rotated in the axial direction by 180° and held for 10 s. A 31-gauge needle was inserted, parallel to the endplates through the AF into the NP, 1.5 mm into the disc to depressurize the nucleus. For the rats in the sham group, the C6–C7 level discs were exposed without needle puncture. The antagomir-195 and antagomir-control were injected into disc C6–C7 of the rats in the punctured + antagomir-195 and punctured + antagomir-195, respectively [11]. The antagomir-195 and antagomir-control were designed and synthesized by RiboBio (Guangzhou, China). After 4 weeks, all rats were sacrificed by lethal anesthetic overdose and discs were collected and used for further analysis.

### Radiography examination

All rats underwent radiography immediately before the IVDD puncture and 4 weeks after the second injection. The disc height index (DHI) was measured using the method as previously described [18]. Changes in the DHI of the punctured IVDDs were expressed as %DHI (%DHI = post-punctured DHI/pre-punctured DHI × 100%).

### HE and TUNEL staining

The paraffin-embedded tissues were cut into 4-μm sections and mounted on slides. The paraffin sections were deparaffinized with xylol and rehydrated. For clearing endogenous peroxidase, the intervertebral discs were treated with PBS containing 0.3% H_2_O_2_ for 15 min, and PBST with 1% BSA was applied to block the sample for 30 min. At last, hematoxylin and eosin (HE) staining was applied onto the samples of intervertebral discs. Terminal deoxynucleotidyl transferase dUTP nick end labeling (TUNEL) staining was performed using a kit based on the manufacturer’s instruction (Promega, Fitchburg, WI, USA). Histological images were analyzed using the BX53 microscope (Olympus Inc., Tokyo, Japan). The grading score of HE staining was made according to the previous study [19].

### Statistical methods

All statistical data were analyzed using SPSS 21.0 (SPSS, Inc., Chicago, IL, USA). Measurement data were presented by mean ± standard deviation. Comparison between two groups was analyzed using Student’s *t* test, while difference among multiple groups was compared using one-way ANOVA followed by a Turkey post hoc test for multiple group comparison. *P* < 0.05 was regarded as statistical significance.

## Results

### Expression of miR-195 and Bcl-2 in NP tissues of IVDD patients

**Fig. 2:**
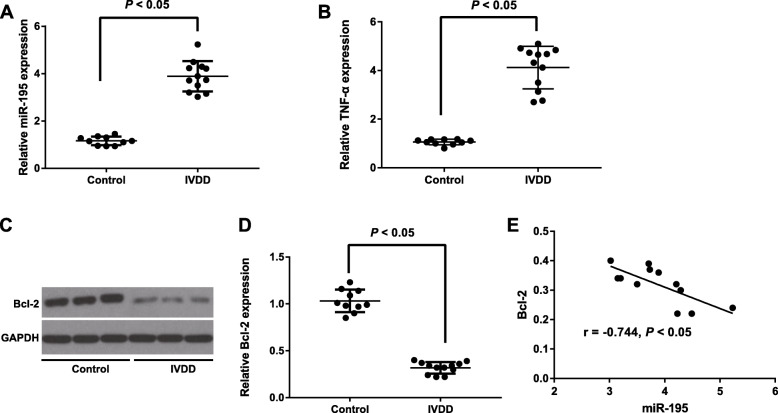
Expression of miR-195 and Bcl-2 in NP tissues of IVDD patients. **A** Expression of miR-195 in NP tissues of IVDD patients and control patients determined by qRT-PCR. **B**, **C** Bcl-2 protein expression in NP tissues of IVDD patients and control patients evaluated by western blotting. **D** Correlation analysis of relationship between miR-195 expression and Bcl-2 expression in NP tissues of IVDD patients

The expressions of miR-195 and TNF-α in NP tissue of IVDD patients and control patients were detected by qRT-PCR (Fig. 2A, B). As a result, the miR-195 and TNF-α expression in NP tissues were significantly higher from IVDD patients than those from the control participants (both *P* < 0.05). Western blotting was performed to detect Bcl-2 protein expression in NP tissues of IVDD patients (Fig. 2C, D). Obviously, IVDD patients had lower Bcl-2 protein level than control participants (*P* < 0.05). As shown in Fig. 2E, miR-195 expression was negatively correlated with the Bcl-2 expression in NP tissues from IVDD patients (r = − 0.744, *P* < 0.05).

### Targeting relationship between miR-195 and Bcl-2

**Fig. 3:**
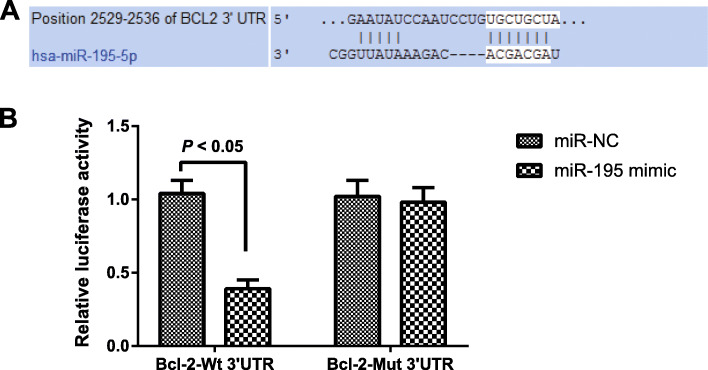
The targeting relationship between miRNA-195 and *Bcl-2*. **A** Biological prediction website (targetscan.org) shows a targeted binding site between miRNA-195 and *Bcl-2*. **B** The result of dual-luciferase reporter gene assay

Biological prediction website (targetscan.org) showed a targeted binding site between miRNA-195 and Bcl-2 (Fig. 3A). The result of dual-luciferase reporter gene assay was displayed in Fig. 3B. As compared with miR-NC group, co-transfection with miR-195 mimic and Bcl-2-Wt 3′UTR can significantly reduce the luciferase activity (all *P <* 0.05), while the co-transfection of miR-195 mimic and Bcl-2-Mut 3′UTR showed no significant changes in the luciferase activity (all *P >* 0.05). These results indicated that Bcl-2 was a target gene of miR-195.

### Comparison of proliferation and apoptosis of NP cells in each group

**Fig. 4:**
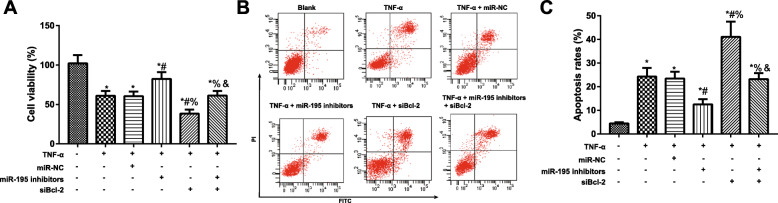
Comparison of the viability and apoptosis of NP cells in each group. **A** Proliferation activity of NP cells detected by MTT assay. **B**, **C** Apoptosis rate of NP cells in each group evaluated by flow cytometry; *P < 0.05 compared with blank group; ^#^*P* < 0.05 compared with TNF-α group; ^%^*P* < 0.05 compared with TNF-α + miR-195 inhibitors group; ^&^*P* < 0.05 compared with TNF-α + siBcl-2 group

As shown in Fig. 4, NP cells in the TNF-α group and TNF-α + miR-NC group had decreased proliferation activity and increased apoptosis rate compared to the blank group (all *P* < 0.05). In comparison with TNF-α group, cells in the TNF-α + miR-195 inhibitors group had significantly increased proliferation activity but apparently decreased apoptosis rate, while those in the TNF-α + siBcl-2 group had declined proliferation and elevated apoptosis (all *P* < 0.05). Besides, compared with TNF-α + miR-195 inhibitors group, TNF-α + miR-195 inhibitors + siBcl-2 group was also strikingly reduced in cell proliferation activity and markedly elevated in cell apoptosis (all *P* < 0.05).

### Comparison of the MMP of NP cells

**Fig. 5:**
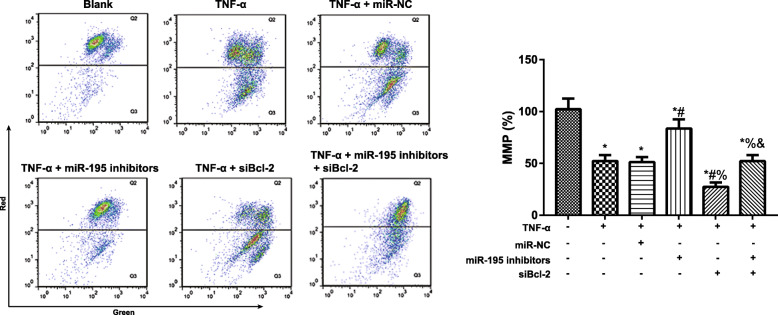
Comparison of the mitochondrial membrane potential (MMP) of NP cells detected by JC-1 staining. *P < 0.05 compared with blank group; ^#^*P* < 0.05 compared with TNF-α group; ^%^*P* < 0.05 compared with TNF-α + miR-195 inhibitors group; ^&^*P* < 0.05 compared with TNF-α + siBcl-2 group

As seen from Fig. 5, compared with the blank group, TNF-α group and TNF-α + miR-NC group declined dramatically in MMP (both *P* < 0.05), but TNF-α group and TNF-α + miR-NC group had no obvious difference in MMP (*P* > 0.05). Compared with TNF-α group, TNF-α + miR-195 inhibitors group was increased appreciably in MMP, while TNF-α + siBcl-2 group was decreased in MMP (all *P* < 0.05). In addition, in comparison with TNF-α + miR-195 inhibitors group, TNF-α + miR-195 inhibitors + siBcl-2 group also declined substantially in MMP (*P* < 0.05).

### Expression of miR-195 and Bcl-2 in NP cells

**Fig. 6:**
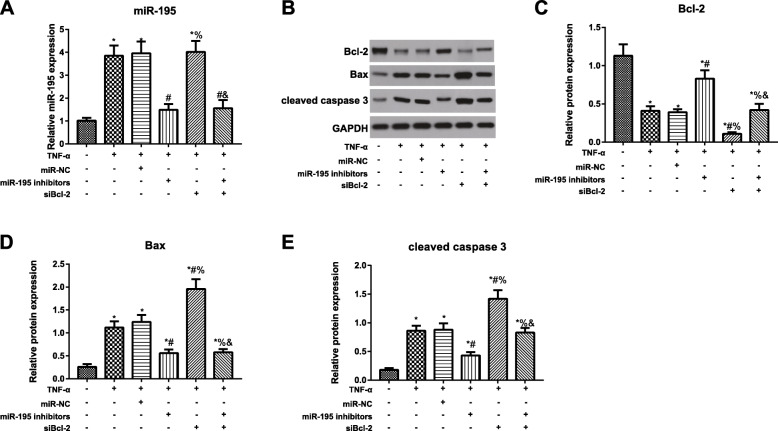
Expression of miR-195 and Bcl-2 in NP cells. **A** MiR-195 expression in NP cells of each group detected by qRT-PCR. **B**–**E** Protein expression of Bcl-2, Bax, and cleaved caspase 3 in NP cells evaluated by western blotting; *P < 0.05 compared with blank group; ^#^*P* < 0.05 compared with TNF-α group; %*P* < 0.05 compared with TNF-α + miR-195 inhibitors group; ^&^*P* < 0.05 compared with TNF-α + siBcl-2 group

As demonstrated in Fig. 6, compared with the blank group, TNF-α group and TNF-α + miR-NC group had the increased expression of miR-195 and Bax and cleaved caspase 3 proteins, but declined Bcl-2 protein expression (all *P* < 0.05). Compared with TNF-α group, TNF-α + miR-195 inhibitors group was significantly downregulated in miR-195, Bax, and cleaved caspase 3 proteins, with the upregulated Bcl-2 protein (all *P* < 0.05), while TNF-α + siBcl-2 group showed no significant difference regarding miR-195 expression level (*P* > 0.05), but decreased in Bcl-2 protein and increased remarkably in Bax and cleaved caspase 3 proteins (all *P* < 0.05). With TNF-α + miR-195 inhibitors as baseline for comparison, TNF-α + miR-195 inhibitors + siBcl-2 group presented no observable difference in miR-195 expression in cells (*P* > 0.05), but showed a significant reduction in Bcl-2 protein and apparent enhancements in Bax and cleaved caspase 3 proteins (both *P* < 0.05).

### MiR-195 inhibition alleviated IVDD in a rat model

**Fig. 7:**
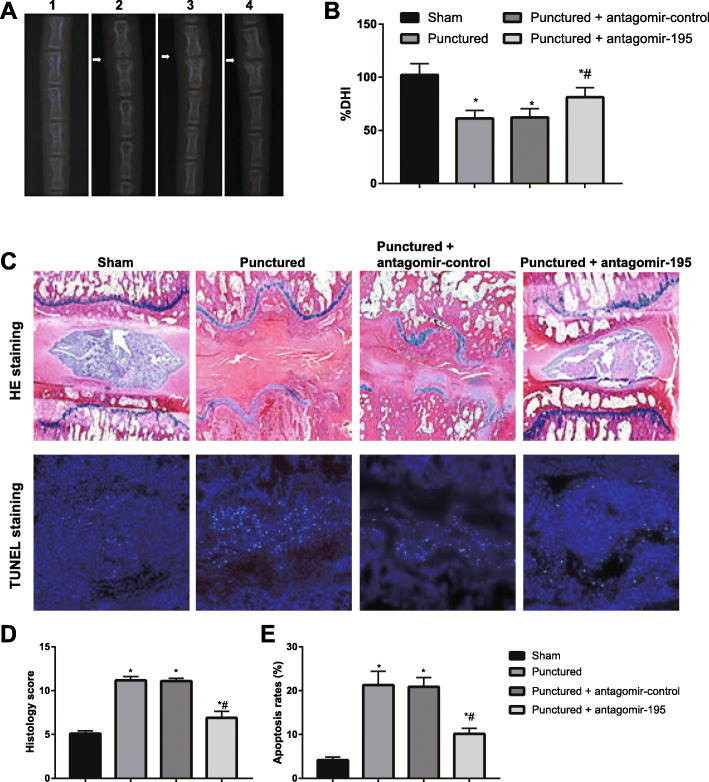
Inhibition of miR-195 attenuated the progression of IVDD in a puncture-induced rat model. **A** Radiographs of X-ray were obtained at 4 weeks post-surgery (the white arrow: location of the needle-puncture disc). **B** The changes in disc height index (DHI) were determined in different groups at 4 weeks post-surgery. **C** Representative HE and TUNEL staining of disc samples from different experimental groups at 4 weeks post-surgery were shown; blue fluorescence indicating total cells; green fluorescence indicating TUNEL-positive cells. **D**, **E** The histology score (D) and NP cell apoptosis rates (**E**) evaluated at 4 weeks post-surgery in different groups; *P < 0.05 compared with sham group; ^#^*P* < 0.05 compared with punctured group

A puncture-induced IVDD model was established in rats to evaluate the therapeutic effects of miR-195 inhibition on IVDD in vivo. X-ray images were taken and the results shown that DHI of punctured discs in punctured group was significantly decreased comparing to sham group, while DHI in punctured + antagomir-195 group was higher than that in the punctured group (all *P* < 0.05, Fig. 7A, B). Based on HE and TUNEL staining (Fig. 7C–E), it was observed that the histology score and NP cell apoptosis rates were remarkably increased in the punctured group compared to the sham group (all *P* < 0.05). However, the punctured + antagomir-195 group showed lower histology score and NP cell apoptosis rates compared with the punctured group (all *P* < 0.05).

## Discussion

In the first place, we observed significant miR-195 upregulation and Bcl-2 downregulation in NP tissues of IVDD patients. The elevated miR-195-5p was also observed in age-related macular degeneration (AMD) by using miRNA microarray chip detection, which was regarded as a potential biomarker for AMD diagnosis, as indicated by Chengda Ren et al. [20]. On the other aspect, Bcl-2 expression was found to be dramatically downregulated, whereas Bax expression was remarkably upregulated in the rabbit IVDD model [21]. Notably, Ping Cai et al. identified the increased expression of miR-15a, which belonged to the same family as miR-195, in NP tissues of IVDD, and in particular, over-expressed miR-15a can inhibit the Bcl-2 expression to promote the apoptosis of NP cells [22]. Although miR-195 demonstrated different targets and functioned in different tissues or diseases, many studies pointed out a pathogenic role of miR-195 in inhibiting cell proliferation and promoting cell apoptosis [23–25]. More importantly, miR-195 expression was found negatively correlated with the Bcl-2 expression in our research, and the dual-luciferase reporter gene assay confirmed Bcl-2 to be a target gene of miR-195. At the same time, downregulation of miR-195 can inhibit ischemic cardiac apoptosis by targeting Bcl-2 [26]. All these findings suggested that miR-195 can regulate the expression of its target gene Bcl-2 to promote the progression of IVDD.

The presence of a significant number of senescent cells were shown in degenerative nucleus pulposus, which can induce the expression of multiple inflammatory cytokines and matrix degrading enzymes to aggravate the living environment of NP cells, thereby affecting the growth and function of NP cells and further triggering the apoptosis of more cells [27]. In fact, TNF-α is a multifunctional pro-inflammatory cytokine and considered to be a key factor in IVDD [28]. As such, we constructed TNF-α-induced NP cell apoptosis model by referring to a previous study [29], aiming to observe the mechanism of miR-195 in inducing NP cell apoptosis in vitro. Firstly, we found that the proliferation of TNF-α-induced NP cells was dramatically decreased, while the cell apoptosis was apparently increased, which was in consistency with the previous findings [30, 31]. Besides, inhibiting miR-195 effectively enhanced the proliferation and limited the apoptosis of TNF-α-induced NP cells, whereas inhibiting Bcl-2 promoted NP cell apoptosis. Similarly, miR-199 was exhibited by Wei Wang et al. to mitigate TNF-α-induced NP cell apoptosis by targeted downregulation of MAP3K5, and thereby playing its protective role for NP cells [32]. Also, HOTAIR can inhibit miR-34a expression to up-regulate the expression of its target gene Bcl-2 to further attenuate TNF-α-induced NP cell apoptosis [33]. Here in this study, Bcl-2 siRNA can reverse the protective effect of miR-195 inhibitor on NP cells. Not surprisingly, Huaqing Zhu et al. noted that miR-195 may reduce Sirt1 and Bcl-2 expression to enhance the reactive oxygen species production and promote the apoptosis of palmitate-induced cardiomyocytes [34]. Taken together, inhibiting miR-195 can inhibit TNF-α-induced NP cell apoptosis by targeted regulation of its target gene Bcl-2.

As for cell apoptosis in IVDD, it can occur through death receptor pathway, mitochondrial pathway and endoplasmic reticulum signaling pathway [35], while its apoptosis induced by endogenous pathway was initially found in mitochondria, namely mitochondrial pathway, which mainly exerts functions via Bcl-2 protein family [36]. As a major anti-apoptotic member, Bcl-2 can maintain the outer mitochondrial membrane integrity [37]. On the contrary, Bax, a pro-apoptotic member of the Bcl-2 family of proteins, mainly plays its regulatory role by destroying the integrity of mitochondrial membrane [38]. Bcl-2 protein family can change the permeability of mitochondrial membrane and induce the opening of mitochondrial pore, thus allowing the apoptosis-inducing factor, cytochrome C, and pro-apoptotic protein into the cytoplasm, and finally inducing cell apoptosis by activating caspase [39, 40]. In agreement, we also found downregulated miR-195 can improve MMP of TNF-α-induced NP cells, elevate Bcl-2 protein, and reduce Bax and cleaved caspase 3 proteins, which however can be reversed by silencing Bcl-2. Besides, inhibition of miR-494 can effectively reduce Caspase-3 and Bax, and elevate Bcl-2, thus promoting the proliferation and hindering the apoptosis of NP cells [41]. As reported by Ping Liu et al., miRNA-125a can upregulate anti-apoptotic protein Bcl-2 and inhibit Caspase-3 and Bax proteins by downregulating BAK1, thus inhibiting the apoptosis of NP cells [42]. Moreover, Chang-Kui Gao et al. demonstrated that down-expressed miR-195 can elevate Bcl-2 but reduce Bax, Cyt-c, and caspase-3 to increase the MMP of cardiomyocytes and mitigate hypoxia re-oxygenation-induced cardiomyocyte apoptosis [43]. Given the above, inhibiting miR-195 can specifically upregulate Bcl-2 to mediate mitochondrial apoptosis pathway, thus playing a protective role to reduce TNF-α-induced NP cell apoptosis. However, there was a limitation in this study. Due to resource limitations, the sample size of patients in the study is small. Thus, investigation with more study patients should be performed to clearly determine the relationships between miR-195 and prognosis of IVDD patients.

To sum up, we observed the increased miR-195 expression in NP tissues of IVDD patients, and inhibition of miR-195 could attenuate TNF-α-induced NP cells apoptosis by upregulating Bcl-2 expression. This study provides a new pathway and scientific basis for the clinical gene-based diagnosis, prevention, and treatment of IVDD.

## Data Availability

The datasets used and/or analyzed during the current study are available from the corresponding author on reasonable request.

## Abbreviations

IVDD: Intervertebral disc degeneration
NP: Nucleus pulposus
MMP: Mitochondrial membrane potential
MiRNA: MicroRNAs
FBS: Fetal bovine serum
NC: Negative control
PVDF: Polyvinylidene fluoride
HRP: Horseradish peroxidase
DMSO: Dimethylsulfoxide
